# Supplementary material for: Shallow subsurface heat recycling is a sustainable global space heating alternative
Source: Nat Commun. 2022 Jul 8;13:3962. doi: 10.1038/s41467-022-31624-6 (PMC9270455; doi:10.1038/s41467-022-31624-6)
Supplement: Supplementary file 1 — Supplementary Information [file 41467_2022_31624_MOESM1_ESM.pdf]

Supplementary Information for

Shallow subsurface heat recycling is a sustainable global space heating alternative

Susanne A. Benz[1*], Kathrin Menberg[2], Peter Bayer[3], Barret L. Kurylyk[1]

[1]Dalhousie University, Centre for Water Resources Studies, Halifax, Canada
[2]Karlsruhe Institute of Technology, Institute of Applied Geosciences, Karlsruhe, Germany
[3]Martin Luther University Halle-Wittenberg, Applied Geology, Halle, Germany
[*]Corresponding authors: susanne.benz@dal.ca and barret.kurylyk@dal.ca

This supplement contains:

- Supplementary Notes (3 pages)
- Supplementary Figures 1-9
- Supplementary Tables 1-6

Supplementary Notes: Details of data availability

We make all data used in this study available in the Scholars Portal Dataverse under `https://doi.org/10.5683/SP3/2UTTVQ` (1). This includes a shapefile and 1 km × 1 km image (.tif) of our classification results (Fig. 5). For those pixels or shapes, values (labels) of 0 indicate class 0: *not feasible*; values of 1 indicate class 1: *potentially feasible*; and values of 2 indicate class 2: *likely feasible*. For the image all other pixels have the value -1. Shared data also includes a table (Data.csv) containing all relevant input variables and results. Each row represents one groundwater measurement location. Each column represents a variable as described below:

- **ID**: Identifies each location. First two letters indicate country, the following symbols the original ID given for this location.
- **lat**: Latitude.
- **lon**: Longitude.
- **mGWT**: (Mulit-)annual mean groundwater temperatures in °C.
- **DeltaT**: Groundwater temperature anomaly ΔT in °C.
- **cV_min**: Estimated min heat capacity of the aquifer in $MJm^{-3}K^{-1}$.
- **cV_median**: Estimated median heat capacity of the aquifer in $MJm^{-3}K^{-1}$.
- **cV_max**: Estimated max heat capacity of the aquifer in $MJm^{-3}K^{-1}$.
- **Q_min**: Min accumulated heat in MJm^{-3}. (Scenario *status quo.*)
- **Q_median**: Median accumulated heat in MJm^{-3}. (Scenario *status quo.*)
- **Q_max**: Max accumulated heat in MJm^{-3}. (Scenario *status quo.*)
- **rho_bld**: estimated building density in percent.
- **lambda_min**: Estimated min thermal conductivity of the unsaturated zone in $Wm^{-1}K^{-1}$.
- **lambda_median**: Estimated median thermal conductivity in $Wm^{-1}K^{-1}$.
- **lambda_max**: Estimated max thermal conductivity in $Wm^{-1}K^{-1}$.
- **GST_min**: Min ground surface temperature in °C.
- **GST_median**: Median ground surface temperature in °C.
- **GST_max**: Max ground surface temperature in °C.
- **GWTable_min**: Min depth to the groundwater table in m.
- **GWTable_median**: Median depth to the groundwater table in m.
- **GWTable_max**: Max depth to the groundwater table in m.
- **qstatusquo_min**: Min heat exchange in MJm^{-2}. (Scenario *status quo.*)

- **qstatusquo_median**: Median heat exchange in MJm^{-2}. (Scenario *status quo*.)
- **qstatusquo_max**: Max heat exchange in MJm^{-2}. (Scenario *status quo*.)
- **qrecycled_min**: Min heat exchange in MJm^{-2}. (Scenario *recycled*.)
- **qrecycled_median**: Median heat exchange in MJm^{-2}. (Scenario *recycled*.)
- **qrecycled_max**: Max heat exchange in MJm^{-2}. (Scenario *recycled*.)
- **GST_rcp45_min**: Min ground surface temperature at the end of the century following RCP4.5 in °C.
- **GST_rcp45_median**: Median ground surface temperature at the end of the century following RCP4.5 in °C.
- **GST_rcp45_max**: Max ground surface temperature at the end of the century following RCP4.5 in °C.
- **GST_rcp85_min**: Min ground surface temperature at the end of the century following RCP8.5 in °C.
- **GST_rcp85_median**: Median ground surface temperature at the end of the century following RCP8.5 in °C.
- **GST_rcp85_max**: Max ground surface temperature at the end of the century following RCP8.5 in °C.
- **q_rcp45_min**: Min heat exchange at the end of the century following RCP4.5 in MJm^{-2}. (Scenario *climate change*.)
- **q_rcp45_median**: Median heat exchange at the end of the century following RCP4.5 in MJm^{-2}. (Scenario *climate change*.)
- **q_rcp45_max**: Max heat exchange at the end of the century following RCP4.5 in MJm^{-2}. (Scenario *climate change*.)
- **q_rcp85_min**: Min heat exchange at the end of the century following RCP8.5 in MJm^{-2}. (Scenario *climate change*.)
- **q_rcp85_median**: Median heat exchange at the end of the century following RCP8.5 in MJm^{-2}. (Scenario *climate change*.)
- **q_rcp85_max**: Max heat exchange at the end of the century following RCP8.5 in MJm^{-2}. (Scenario *climate change*.)
- **HDD_min**: Min heating degree days based in °C.
- **HDD_median**: Median heating degree days based in °C.
- **HDD_max**: Max heating degree days based in °C.
- **GDP**: GDP per capita, PPP in 2017 US$.
- **HeatIntensity**: Space heating intensity in KJm^{-2}.

- **population_min**: Min population in km^{-2}.
- **population_median**: Median population in km^{-2}.
- **population_max**: Max population in km^{-2}.
- **Qdemand_min**: Min heating demand in MJm^{-2}.
- **Qdemand_median**: Median heating demand in MJm^{-2}.
- **Qdemand_max**: Max heating demand in MJm^{-2}.
- **HDD_rcp45_min**: Min heating degree days at the end of the century following RCP4.5 based in °C.
- **HDD_rcp45_median**: Median heating degree days at the end of the century following RCP4.5 based in °C.
- **HDD_rcp45_max**: Max heating degree days at the end of the century following RCP4.5 based in °C.
- **HDD_rcp85_min**: Min heating degree days at the end of the century following RCP8.5 based in °C.
- **HDD_rcp85_median**: Median heating degree days at the end of the century following RCP8.5 based in °C.
- **HDD_rcp85_max**: Max heating degree days at the end of the century following RCP8.5 based in °C.
- **pop_SSP2**: Population at the end of the century following SSP2 in km^{-2}.
- **pop_SSP5**: Population at the end of the century following SSP5 in km^{-2}.
- **Qdemand_rcp45_min**: Min heating demand at the end of the century following RCP4.5 and SSP2 in MJm^{-2}.
- **Qdemand_rcp45_median**: Median heating demand at the end of the century following RCP4.5 and SSP2 in MJm^{-2}.
- **Qdemand_rcp45_max**: Max heating demand at the end of the century following RCP4.5 and SSP2 in MJm^{-2}.
- **Qdemand_rcp85_min**: Min heating demand at the end of the century following RCP8.5 and SSP5 in MJm^{-2}.
- **Qdemand_rcp85_median**: Median heating demand at the end of the century following RCP8.5 and SSP5 in MJm^{-2}.
- **Qdemand_rcp85_max**: Max heating demand at the end of the century following RCP8.5 and SSP5 in MJm^{-2}.

Supplementary Figures

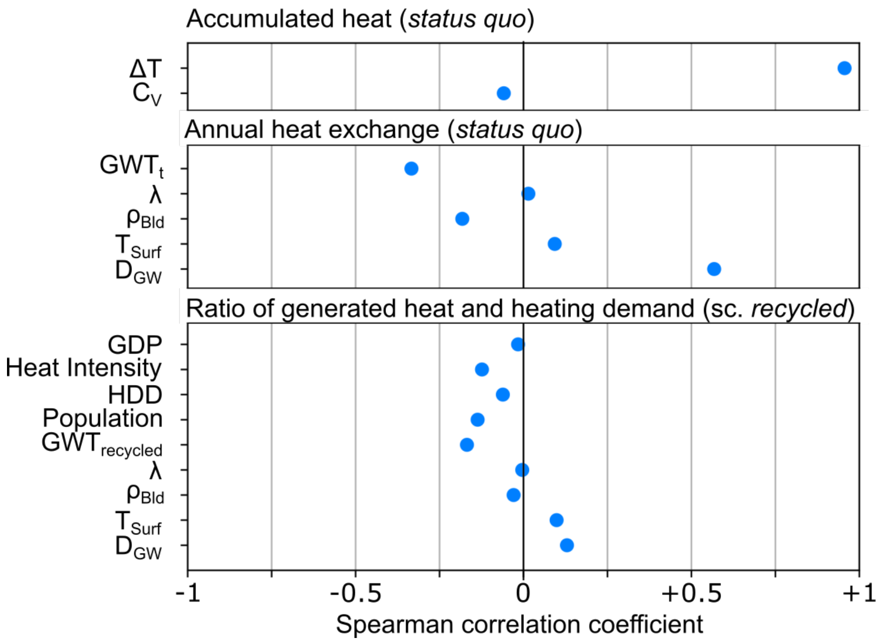

Supplementary Figure 1: **Contribution-to-variance of input variables and results.** Spearman correlation coefficients between variables given on the left and top. For the accumulated heat of scenario *stats quo* we analysed the correlation to ΔT and heat capacity c_V; for the annual heat exchange in the *stats quo* we analysed the correlation to GWTs, the thermal conductivity λ, building density ρ_{Bld} (eq. 3), ground surface temperatures T_{Surf}, and groundwater table depth D_{GW}; Lastly for the ratio of generated heat (scenario *recycled*) and heating demands we show the correlation to GDP, heat intensity, population, $GWT_{recycled}$ (eq. 7), the thermal conductivity λ, building density ρ_{Bld} (eq. 3), ground surface temperatures T_{Surf}, and groundwater table depth D_{GW}.

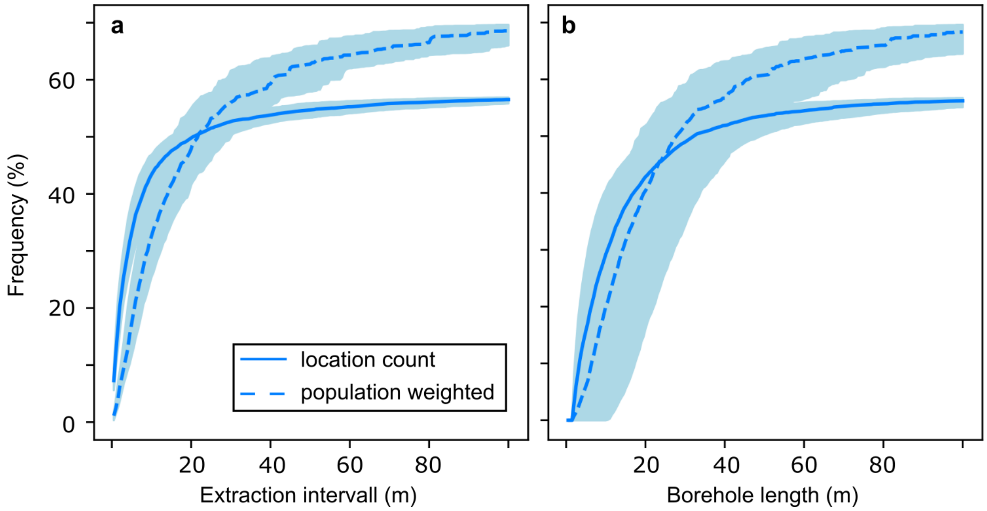

Supplementary Figure 2: **Extraction interval and borehole depth needed to supply one year of heat from accumulated heat.** This analysis is based only on locations with unconsolidated sediments. Histogram of the required **a**: extraction interval and **b**: borehole depth (= extraction interval + depth to the groundwater table) to ensure that the generated thermal energy from recycling the accumulated heat is equal to the local annual space heating demand.

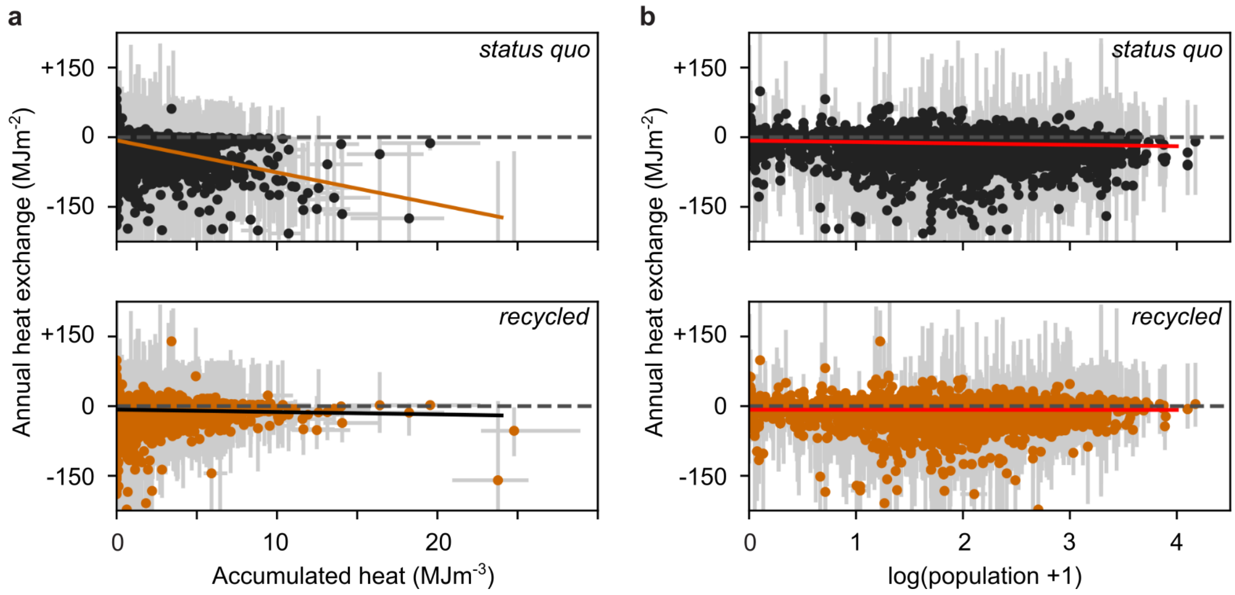

Supplementary Figure 3: **Comparing heat exchange, accumulated heat, and population density.** **a**: Link between heat exchange and accumulated heat for the *status quo* and *recycled* scenarios. **b**: Link between heat exchange and population density for the *status quo* and *recycled* scenarios.

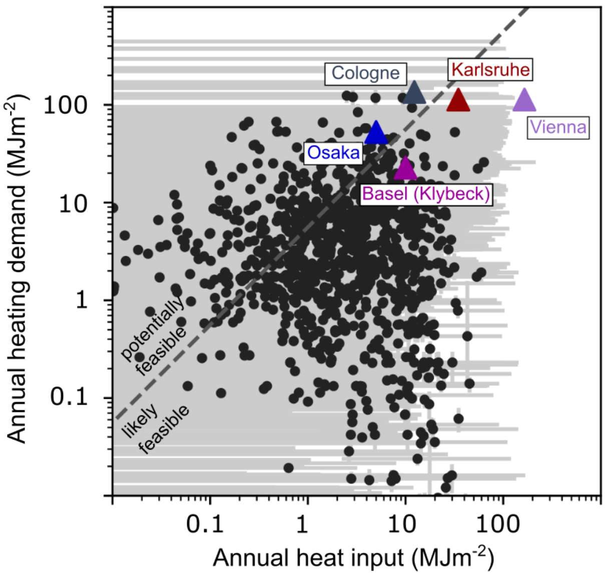

Supplementary Figure 4: **Comparison of annual mean heat input of our scenario *status quo* and heating demand**. Only locations with unconsolidated sediments are shown. The diagonal line separates *likely feasible* locations from *potentially feasible* locations. Triangles indicate results from previous publications (2–6). (Note that locations with no heating demand or no heat input are not shown).

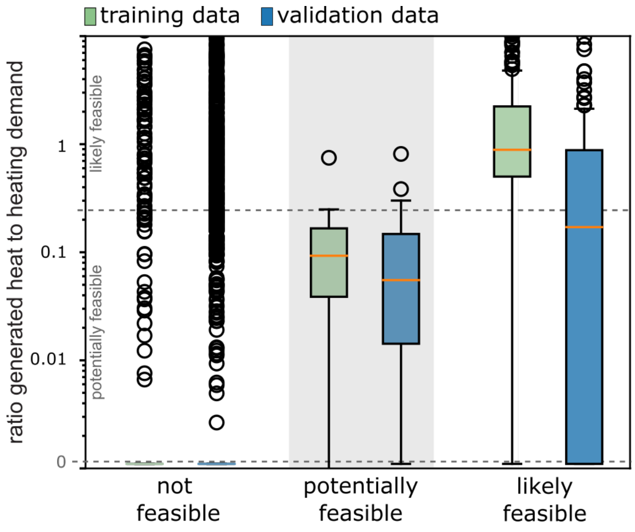

Supplementary Figure 5: **Accuracy assessment of the random forest classification.** The y-axis shows the observed ratio of generated thermal energy when recycling the annual heat input and heating demands, while the x-axis indicates classification results. Green bars represent all locations used for training, blue bars locations used for validation only.

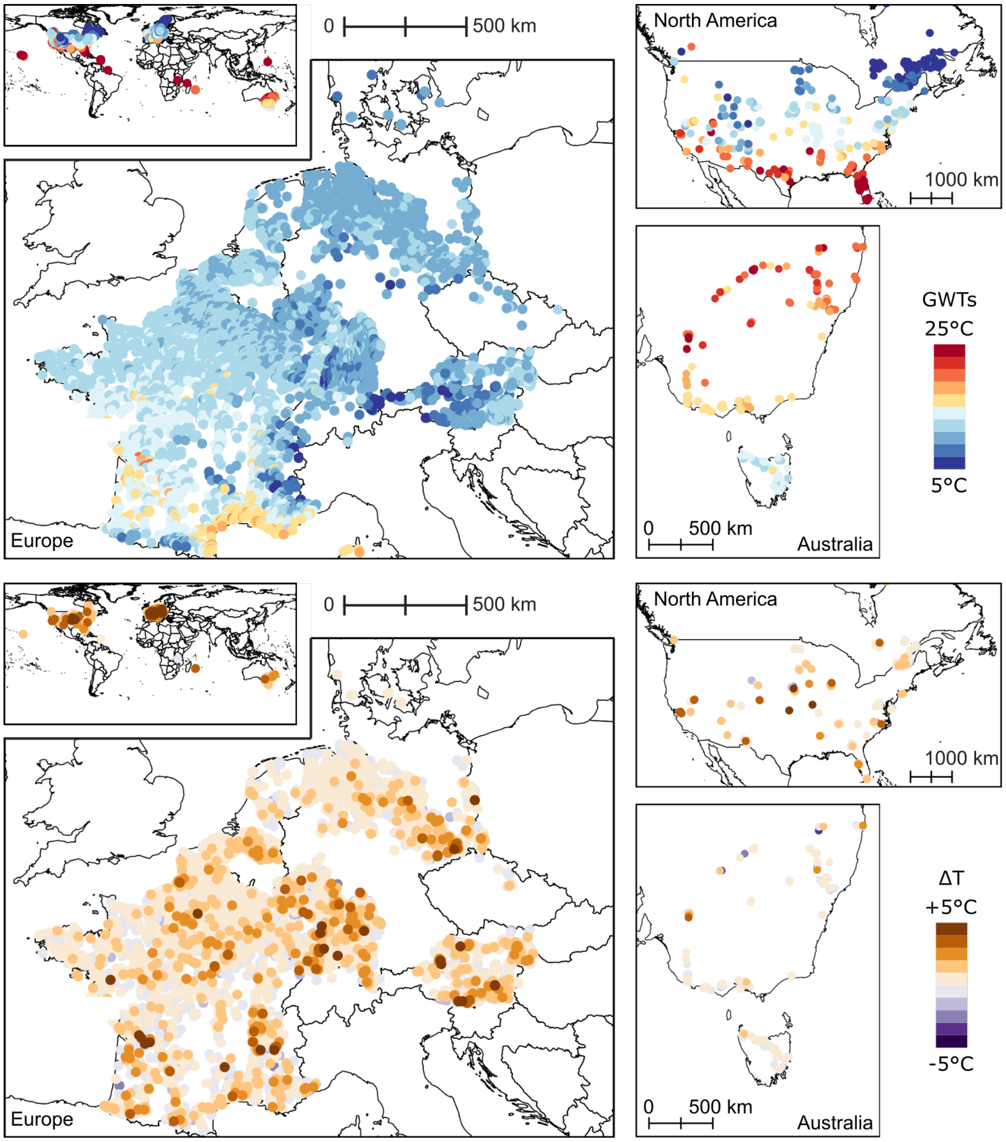

Supplementary Figure 6: **Maps of (multi-) annual mean groundwater temperatures (GWT) and their anomalies ΔT.**

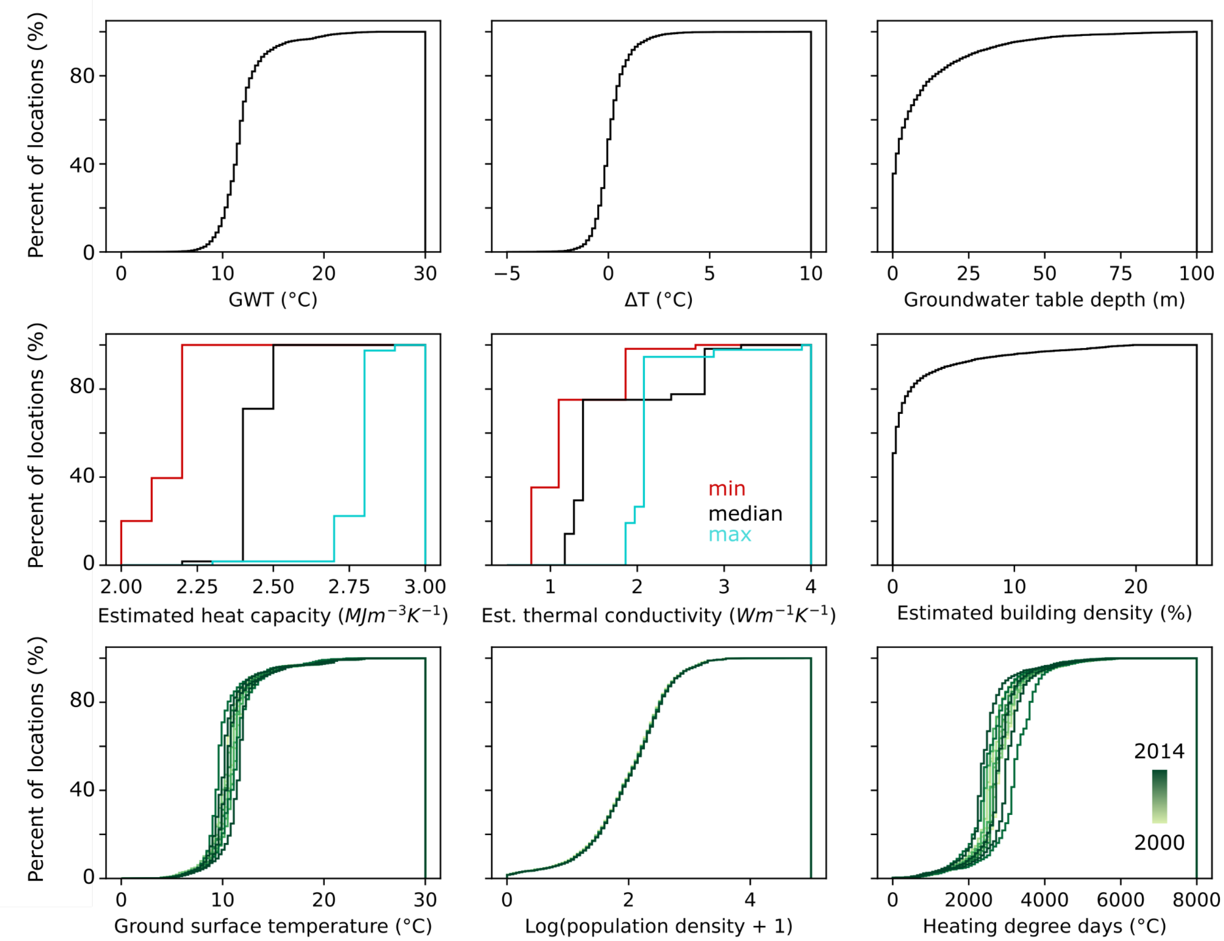

Supplementary Figure 7: **Histograms of various input parameters**: groundwater temperatures (GWTs), their anomalies (ΔT), groundwater table depth, estimated heat capacity, estimated thermal conductivity, estimated building density, ground surface temperatures, population density, and heating degree days. Parameters collected in several year are given for each year separately.

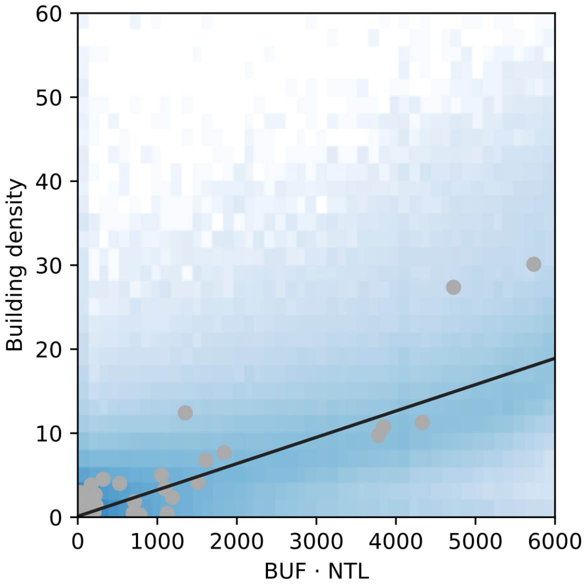

Supplementary Figure 8: **Estimating building density.** Regression between building density (7) and the product of the built-up land cover fraction (BUF) and nighttime lights (NTL). The heat map represents all pixels of the contiguous United States for which building density is available, dots are our groundwater temperature measurement locations in the US. The best fit has a R^2 of 0.73. See Supplementary Table 4 for other fit options.

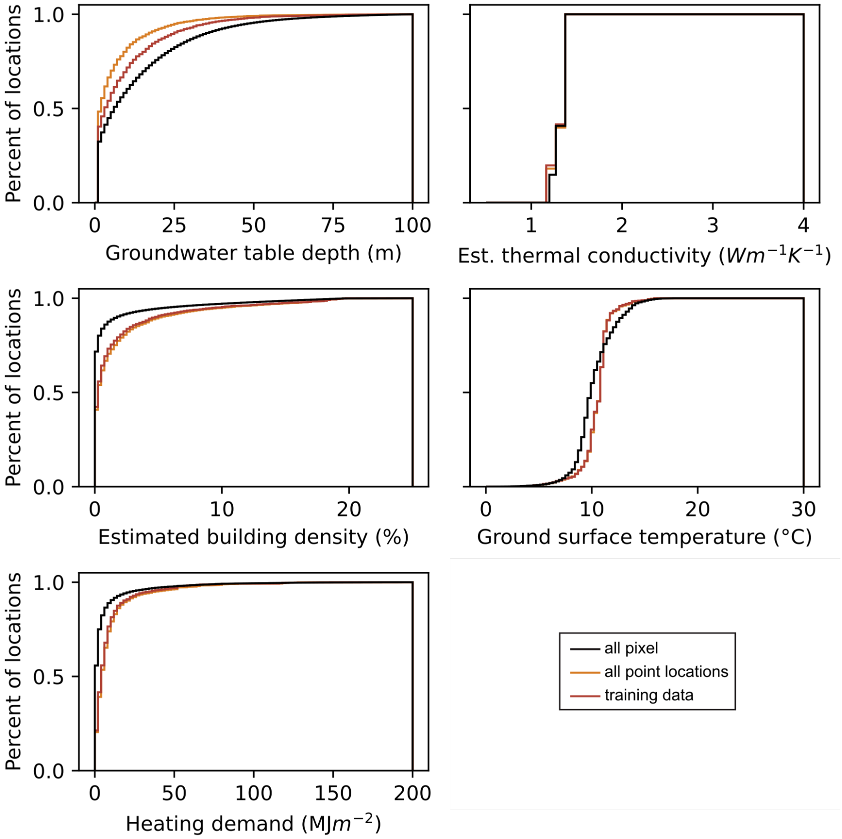

Supplementary Figure 9: **Distribution of variables in training data and pixels to be classified.** Cumulative histograms of all variables used for the random forest classification for all European locations with unconsolidated sediments (orange line), the excerpt of those used to train the random forest classifier (red line), and all pixels on which the classifier has been applied to (black line).

Supplementary Tables

Supplementary Table 1: Assumed values for heat capacity c_V of the aquifer and thermal conductivity λ of the unsaturated zone (where applicable) for our median (min; max) calculations. Values are based on the VDI 4640 (8). Soil types marked with a * indicated that extraction of heat may not be possible due to technical constrains.

grain size (9)	soil type	c_V $(MJm^{-3}K^{-1})$	λ $(Wm^{-1}K^{-1})$	#locations
su	sand and coarser	2.4 (2.2; 2.8)	1.4 (1.1; 2.1)	1,259
sl, sc	sand and silt or clay	2.4 (2.1; 2.8)	1.3 (0.8; 1.9)	1,152
lu, lc, cu	silt and/or clay	2.4 (2.0; 2.8)	1.2 (0.8; 2.1)	1,157
mx	mixed	2.5 (2.0; 2.8)	1.4 (0.8; 2.0)	439
sediment class (9)				
Au, Cu, Ed, Gu, Gf, Gt, Us	sand and coarser	2.4 (2.2; 2.8)	1.4 (1.1; 2.1)	1,412
Al, Ca, Op	sand and silt or clay	2.4 (2.1; 2.8)	1.3 (0.8; 1.9)	85
Wu, Zu	mixed	2.5 (2.0; 2.8)	1.4 (0.8; 2.0)	34
lithology class (10)				
su	sand and coarser	2.4 (2.2; 2.8)	1.4 (1.1; 2.1)	560
ss, sm, sc	sedimentary rock*	2.5 (2.2; 2.7)	2.8 (1.9; 4.6)	1,673
vb, va, vi, pb, pa	magmatic rock*	2.5 (2.1; 2.9)	2.4 (1.9; 2.9)	206
mt	metamorphic rock*	2.2 (2.1; 2.3)	3.2 (2.7; 3.9)	141
wb	mixed	2.5 (2.0; 2.8)	1.4 (0.8; 2.0)	1

Supplementary Table 2: Summery of all groundwater temperature data used in this study. Values are given as median (5th; 95th percentile).

**Europe**	#Locations	GWT	#Observation	Seasonal radius	Source
Austria	1,062	10.8 (7.8; 13.1)	83 (28; 180)	0.02 (0.00; 0.10)	(11, 12)
Belgium, Brussels	16	12.6 (10.1; 14.9)	8 (3; 15)	0.20 (0.00; 0.24)	(13, 14)
Belgium, Flanders	2,047	11.8 (11.1; 12.7)	42 (18; 74)	0.11 (0.03; 0.22)	(14, 15)
Czechia	14	10.6 (9.7; 11.4)	16 (9; 25)	0.21 (0.17; 0.25)	(14, 16)
Denmark	53	9.9 (8.6; 10.5)	78 (63; 119)	0.08 (0.04; 0.13)	(14, 17)
France	3,235	12.3 (10.2; 16.2)	17 (4; 66)	0.13 (0.00; 0.24)	(18, 19)
Germany, Baden-Wurttemberg	646	10.9 (7.9; 13.4)	37 (18; 51)	0.06 (0.00; 0.23)	(12, 20)
Germany, Brandenburg	44	10.6 (9.2; 12.9)	1,015 (486; 1,904)	0.08 (0.01; 0.15)	(14, 21)
Germany, Hesse	4	12.0 (10.0; 14.4)	812 (350; 1,752)	0.19 (0.04; 0.22)	(14, 22)
Germany, Lower Saxony	507	10.4 (9.4; 11.5)	16 (10; 24)	0.16 (0.05; 0.24)	(14, 23)
Germany, North Rhine-Westphalia	40	10.4 (8.4; 12.2)	1,280 (285; 4,243)	0.07 (0.01; 0.24)	(14, 24)
Germany, Rhineland-Palatinate	259	11.4 (8.7; 13.0)	20 (2; 30)	0.09 (0.00; 0.21)	(14, 25)
Germany, Saarland	30	9.8 (8.6; 13.2)	13,823 (26; 15,390)	0.13 (0.06; 0.22)	(14, 26)
Germany, Saxony	295	10.8 (9.5; 13.4)	14 (2; 30)	0.13 (0.00; 0.24)	(14, 27)
Germany, Saxony-Anhalt	251	10.7 (9.8; 12.3)	13 (9; 19)	0.16 (0.03; 0.24)	(14, 28)
Germany, Thuringia	7	9.4 (7.4; 13.1)	495 (206; 575)	0.04 (0.00; 0.07)	(14, 29)
Luxembourge	107	9.3 (8.7; 10.7)	34 (7; 45,457	0.10 (0.03; 0.20)	(14, 30)
Netherlands	109	11.2 (9.8; 12.5)	5 (2; 12)	0.17 (0.00; 0.25)	(14, 31)
Switzerland	31	11.0 (9.2; 13.4)	3,581 (2,910; 4,383)	0.03 (0.01; 0.06)	(14, 32)

Supplementary Table 3: Cont'd Supplementary Table 1

**Australia**	#Locations	GWT	#Observation	Seasonal radius	Source
Australia, New South Wales	126	20.4 (16.2; 23.4)	1,295 (391; 3,332)	0.07 (0.01; 0.19)	(33)
Australia, Tasmania	75	13.8 (12.4; 14.9)	14,673 (9,521; 29,214)	0.16 (0.04; 0.22)	(34)
Australia, Victoria	138	16.7 (15.5; 21.4)	18 (4; 44)	0.11 (0.02; 0.24)	(35)
**North America**	#Locations	GWT	#Observation	Seasonal radius	Source
Canada, British Columbia	2	12.4 (5.8; 18.9)	7 (3; 11)	0.19 (0.17; 0.20)	(36, 37)
Canada, Quebec	216	7.6 (4.1; 9.1)	1,696 (454; 3,934)	0.05 (0.02; 0.18)	(38)
USA	849	14.7 (9.4; 24.9)	8 (2; 1,999)	0.12 (0.00; 0.24)	(39)
**Africa**	#Locations	GWT	#Observation	Seasonal radius	Source
Tanzania	5	24.3 (23.7; 25.9)	2 (2; 2)	0.00 (0.00; 0.00)	(12, 40)

Supplementary Table 4: Coefficients for all tested multi-linear regressions between building density and population density (log(Pop+1)), built-up fraction (BUF), and nighttime lights (NTL) as well as combinations thereof. Information on the fit are given in the form of root mean square residuals (rmsr) and the coefficient of determination R^2. Best fit was achieved by linking building density to the product of NTL and BUF (marked in bold).

Intercept	log(Pop+1)	BUF	NTL	log(Pop+1) · BUF	NTL · BUF	rmsr	R^2
-0.06	0.70	0.17	0.008	-	-	1.12	0.73
-0.06	0.14	0.17	-	-	-	1.12	0.73
-0.29	0.37	-	0.100	-	-	1.60	0.44
-0.03		0.17	0.011	-	-	1.12	0.73
-0.41	1.60	-	-	-	-	1.77	0.31
-0.00	-	0.18	-	-	-	1.12	0.72
-0.19	-	-	0.116	-	-	1.60	0.43
0.09	-	-	-	0.06	-	1.11	0.73
**0.11**	-	-	-	-	**0.003**	**1.10**	**0.73**

Supplementary Table 5: Assumed values for GDP (41) and useful space heating intensity needed (42, 43) to estimate heating demands. Values marked with * were set equal to Germany, values marked with ** were estimated. Countries marked with * are not represented by our ΔT locations but were part of the random forest classification later on.

Country	GDP per capita, PPP (2017 US$)	Space heating int. ($kJm^{-2}K^{-1}$)
Albania*	11587	160**
Australia	47290	130
Austria	52857	150*
Belgium	48748	150*
Bosnia and Herzegovina*	12067	160**
Canada	47565	90
Croatia*	23783	160**
Czechia	34387	150*
Denmark	52048	110
France	43021	180
Germany	50771	150
Hungary*	26425	150*
Italy*	39899	160**
Kosovo*	9214	160**
Luxembourg	108415	150*
Montenegro*	17675	160**
Netherlands	52187	150*
Poland*	26650	150*
Serbia*	15226	160**
Slovakia*	27385	150*
Slovenia*	33099	150*
Spain*	35969	160**
Sweden*	49259	110
Switzerland	67683	150*
Tanzania	2285	40**
United Kingdom*	44154	130
USA	57213	90

Supplementary Table 6: Assumed values for the thermal conductivity λ of the unsaturated zone when used in the random forest classification. These values are an addition to Supplementary Table 1 and give values for all sediment classes found in Europe but not our selected ΔT locations. All grain sizes and lithology classes in Europe are represented in our locations and hence stated in Supplementary Table 1.

sediment class (9)	soil type	λ ($Wm^{-1}K^{-1}$)
At, Eu, Yu, Yb, Yd, Yl, Ys	sand and coarser	1.4
Af, Er, Gm, Gp, Mu, Ou, Or, Pu	sand and silt or clay	1.3
Ae, Ap, Ea, El, Gl, Lu, Pp, Ym	silt and/or clay	1.2
Wr	mixed	1.4

References

[1] Benz, S. Data for "Waste heat in the shallow underground: an untapped resource for global sustainable heating" (2021). URL https://doi.org/10.5683/SP3/2UTTVQ.

[2] Benz, S. A., Bayer, P., Menberg, K., Jung, S. & Blum, P. Spatial resolution of anthropogenic heat fluxes into urban aquifers. *Science of The Total Environment* **524-525**, 427–439 (2015). URL https://doi.org/10.1016/j.scitotenv.2015.04.003.

[3] Benz, S. A. *et al.* Comparing anthropogenic heat input and heat accumulation in the subsurface of Osaka, Japan. *Science of The Total Environment* **643**, 1127–1136 (2018). URL https://doi.org/10.1016/j.scitotenv.2018.06.253.

[4] Tissen, C. *et al.* Identifying key locations for shallow geothermal use in Vienna. *Renewable Energy* **167**, 1–19 (2021). URL https://doi.org/10.1016/j.renene.2020.11.024.

[5] Epting, J., Müller, M. H., Genske, D. & Huggenberger, P. Relating groundwater heat-potential to city-scale heat-demand: A theoretical consideration for urban groundwater resource management. *Applied Energy* **228**, 1499–1505 (2018). URL https://doi.org/10.1016/j.apenergy.2018.06.154.

[6] Mueller, M. H., Huggenberger, P. & Epting, J. Combining monitoring and modelling tools as a basis for city-scale concepts for a sustainable thermal management of urban groundwater resources. *Science of The Total Environment* **627**, 1121–1136 (2018). URL https://doi.org/10.1016/j.scitotenv.2018.01.250.

[7] Heris, M. P., Foks, N. L., Bagstad, K. J., Troy, A. & Ancona, Z. H. A rasterized building footprint dataset for the United States. *Scientific Data* **7** (2020). URL https://doi.org/10.1038/s41597-020-0542-3.

[8] VDI-Gesellschaft Energie und Umwelt (GEU). VDI 4640 - thermal use of the underground (2010).

[9] Börker, J., Hartmann, J., Amann, T. & Romero-Mujalli, G. Terrestrial sediments of the earth: Development of a global unconsolidated sediments map database (GUM). *Geochemistry, Geophysics, Geosystems* **19**, 997–1024 (2018). URL https://doi.org/10.1002/2017gc007273.

[10] Hartmann, J. & Moosdorf, N. The new global lithological map database GLiM: A representation of rock properties at the earth surface. *Geochemistry, Geophysics, Geosystems* **13** (2012). URL https://doi.org/10.1029/2012gc004370.

[11] Abteilung Wasserhaushalt im Bundesministerium für Land- und Forstwirtschaft, Umwelt und Wasserwirtschaft (BMLUFUW) (2016). URL https://ehyd.gv.at/.

[12] Benz, S. A., Bayer, P. & Blum, P. Identifying anthropogenic anomalies in air, surface and groundwater temperatures in Germany. *Science of The Total Environment* **584-585**, 145–153 (2017). URL https://doi.org/10.1016/j.scitotenv.2017.01.139.

[13] Bruxelles Environment (2018). URL https://environnement.brussels/.

[14] Tissen, C., Benz, S. A., Menberg, K., Bayer, P. & Blum, P. Groundwater temperature anomalies in central Europe. *Environmental Research Letters* **14**, 104012 (2019). URL https://doi.org/10.1088/1748-9326/ab4240.

[15] Vlaamse Milieumaatschappij (VMM) (2018). URL https://www.vmm.be.

[16] Czech Hydrometeorological Institute (CHMI) (2018). URL http://hydro.chmi.cz/.

[17] Geological Survey of Denmark and Greenland (GEUS) (2018). URL https://www.geus.dk/.

[18] Portail nationale d'Acces aux Donnees sur les Eaux Souterraines (ADES) (2021). URL https://ades.eaufrance.fr/.

[19] Bureau de Recherches Geologiques et Minieres (BRGM) (2021). URL http://infoterre.brgm.fr/viewer/MainTileForward.do.

[20] Landesamt fuer Umwelt, Messungen und Naturschutz Baden-Wurttember, (LUBW) (2016). URL http://jdkgw.lubw.baden-wuerttemberg.de/servlet/is/200/.

[21] Landesamt fur Umwelt - Brandenburg (LfUBB) (2018). URL https://lfu.brandenburg.de/lfu/de/.

[22] Hessisches Landesamt fuer Naturschutz, Umwelt und Geologie (HLNUG) (2018). URL https://www.hlnug.de/start.html.

[23] Nds. Landesbetrieb fur Wasserwirtschaft, Kuesten- und Naturschutz (NLWKN (2018). URL https://www.nlwkn.niedersachsen.de/startseite/.

[24] Landesamt fur Natur, Umwelt und Verbraucherschutz Nordrhein-Westfalen (LANUV) (2018). URL https://www.lanuv.nrw.de/.

[25] Landesamt fur Umwelt Rheinland-Pfalz (LfURP) (2018). URL https://lfu.rlp.de/de/startseite/.

[26] Landesamt fur Umwelt- und Arbeitsschutz Saarland (LUA) (2018). URL https://www.saarland.de/landesamt_umwelt_arbeitsschutz.htm.

[27] Landesamt fur Umwelt, Landwirtschaft und Geologie Sachsen (LfULG) (2018). URL https://lfulg.sachsen.de/.

[28] Landesbetrieb fur Hochwasserschutz und Wasserwirtschaft Sachsen-Anhalt (LHW) (2018). URL https://lhw.sachsen-anhalt.de/.

[29] Thueringer Landesanstalt fuer Umwelt und Geologie (TLUG) (2018). URL https://www.thueringen.de/th8/tlug/.

[30] Administration de la gestion de l'eau Luxembourg (AGE) (2018). URL https://eau.public.lu/.

[31] Informatiehuis Water (IHW) (2018). URL https://www.ihw.nl/index.html.

[32] Bundesamt fuer Umwelt - Schweiz (BAFU), Nationale Grundwasserbeobachtung (NAQUA) (2018). URL https://www.bafu.admin.ch/bafu/de/home.html.

[33] WaterNSW Water Information Hub (2021). URL https://realtimedata.waternsw.com.au/.

[34] Water Information Tasmania Web Portal (2021). URL https://portal.wrt.tas.gov.au/.

[35] Water Measurement Information System, Department of Environment, Land, Water and Planning (DELWP), Victoria (2021). URL https://data.water.vic.gov.au/static.htm.

[36] Government of British Columbia, Groundwater Wells and Aquifers (2021). URL https://apps.nrs.gov.bc.ca/gwells/.

[37] Government of British Columbia, Data search & download (2021). URL https://a100.gov.bc.ca/pub/ems/dataResultsCriteria.do.

[38] Ministère de l'Environnement et de la Lutte contre les changements climatiques (MDDELCC) (2021). URL https://www.environnement.gouv.qc.ca/eau/piezo/index.htm.

[39] USGS Groundwater Data for the Nation (2021). URL https://waterdata.usgs.gov/nwis/gw.

[40] Delalande, M. *Hydrologie et geochimie isotopique du lac masoko et de lacs volcaniques de la province active du rungwe (sud-ouest Tanzanie)*. Ph.D. thesis, Université Paris Sud (2008).

[41] WorldBank. GDP per capita, PPP (constant 2017 international $) (ny.gdp.pcap.pp.kd). URL https://databank.worldbank.org/reports.aspx?source=2\&series=NY.GDP.PCAP.PP.KD\&country=.

[42] IEA. Oil crises and climate challenges: 30 years of energy use in iea countries (2004). URL https://www.iea.org/reports/oil-crises-and-climate-challenges-30-years-of-energy-use-in-iea-countries.

[43] Isaac, M. & van Vuuren, D. P. Modeling global residential sector energy demand for heating and air conditioning in the context of climate change. *Energy Policy* **37**, 507–521 (2009). URL https://www.sciencedirect.com/science/article/pii/S0301421508005168.
